# Role of community based savings groups (CBSGs) enhancing the utilization of community midwives in chitral district of Pakistan

**DOI:** 10.1186/1471-2393-13-185

**Published:** 2013-10-11

**Authors:** Qayyum Ali Noorani, Iqbal Azam, Babar T Shaikh, Tharanga Ranasinghe, Shazia Abbas, Shakeela Wali, Paul Rippey, Wajiha Javed

**Affiliations:** 1 Aga Khan Foundation, Islamabad, Pakistan; 2 CHS Department, Aga Khan University, Karachi, Pakistan; 3 Health Services Academy, Islamabad, Pakistan; 4 Aga Khan Rural Support Program, Gilgit, Pakistan; 5 George Washington University, Washington DC, USA

**Keywords:** Community based savings groups, Community midwives, Maternal Neonatal and Child Health

## Abstract

**Background:**

Maternal and infant mortality rates in the district of Chitral in Pakistan are alarmingly high. One of the major reasons for this is the inability of women to access skilled care due to the high costs associated with traveling and utilizing such services. The Aga Khan Health Services, Pakistan (AKHSP) in partnership with the national and provincial Maternal, Neonatal and Child Health (MNCH) program, deployed 28 community midwives (CMWs) in remote villages of Chitral district. This program has also established Community-Based Savings Groups (CBSGs) to support and facilitate access to MNCH services, in particular those delivered by the CMWs. CBSGs are a simple yet cost-effective and sustainable means of providing basic financial services to low income, marginalized, rural populations.

The link between CBSGs and utilization of MNCH services is not well understood. This study will assess the relationship between women membership of CBSGs and their utilization of MNCH services, specifically those offered by CMWs, in the community.

**Methods:**

The research question will be answered through guided interviews of women in the target population who have delivered within one month. The outcome variable will be the utilization of full continuum of skilled MNCH care (disaggregated by 1+ ANC, 1+ PNC and skilled delivery). The primary independent variable of interest will be participation in a CBSG.

Focus Group Discussions (FGDs) will be conducted to generate further understanding and information about the social and financial factors that contribute to health behavior and health provider decision-making during pregnancy.

Analysis will be tailored to answer how CBSGs, directly or indirectly, facilitate greater financial and/or social access to CMW services for pregnant women. Furthermore, the extent to which financial or social empowerment through a CBSG leads to greater utilization of CMW services.

**Discussion:**

The role of CBSGs and their interlink with the CMWs services to be replicated in other comparable areas in Pakistan as a viable mean to increase MNCH service utilization amongst rural, low income, and marginalized communities. Findings from this research will be disseminated through community, national, and international channels consisting of policy makers and social society groups.

## Background

### Context analysis: burden of disease and hindrances

The province of Khyber-Pukhtunkhwa (KP) has one of the highest levels of maternal and infant mortality and morbidity in Pakistan. An estimated 85% of all deliveries take place at home [1] compared with a national average of 65% [2], and these rates continue to escalate. Barriers encountered in seeking maternal and newborn care are long travel distances, lack of transportation, and cost of care [3]. Furthermore, the economic dependence of women on their household leads to a lack of decision-making authority, which impairs their ability to receive healthcare.

Chitral is the largest district with lowest population density in the province of KP. A widely dispersed population, difficult mountainous terrain, harsh weather, and poor road infrastructure makes traveling to health facilities expensive and time consuming. The average distance of a first level health care facility (FLHCF) to a secondary level referral facility (SLRF) is 45 km, and the average health expenditure per person per visit is PKR 1,474 (~ $25) of which 50% is spent on transportation.

According to the baseline survey conducted before launching this project, only 20% of home-based deliveries were assisted by a skilled birth attendant (SBA), comprising of (doctor, nurse, lady health worker, or midwife) and 73.4% of deliveries took place at home. 21.5% of women who did not receive any antenatal care (ANc) stated that the medical facilities were too far. 41% and 18% of the district population was classified as poor and ultra-poor, respectively [4].

It is estimated that 85% of deliveries in a community are normal vaginal deliveries while the remaining 15% require some intervention. About a third of the latter may be treated at the SLRF by Woman Medical Officer (WMOs) through an ‘assisted vaginal’ delivery while the remaining require the services of an obstetrician at an EmONC facility [5]. One of the major obstacles to accessing skilled care, both for normal deliveries and for those requiring emergency interventions, is the inability to bear the costs associated with traveling to and utilizing such services [6].

### Community midwives: a new cadre of community-level health providers

As part of the effort to check the maternal and neonatal mortality and morbidity rates, the Government of Pakistan (GoP) introduced a new cadre of community-level health providers – the CMWs - in the country. The CMWs are to address the lack of medically trained skilled health providers at the community level by acting as skilled alternatives to the traditional unskilled birth assistants in rural communities. They are trained to provide the full spectrum of antenatal and post-natal continuum of care at the community level. In addition to imparting basic family planning advice, CMWs can assist in birth preparedness, provide complication readiness counseling and timely referral to the SLRF.

Considering the rural communities’ preference for home-based deliveries and the difficulties in accessing formal medical facilities because of distance and cost, training and deploying CMWs is likely to increase availability and accessibility of skilled obstetric and neonatal care. Available at the doorstep of pregnant women, the CMWs would allow the mother to remain home and access prenatal, delivery, and postnatal services.

The catchment area of the CMW is determined by the population to be covered, and the travel time on foot from the residence of CMW to the farthest house in the allocated area. The national MNCH guidelines originally estimated that each CMW could cover around 8000 people. However, it has recently been noted that each CMW can realistically only cover clusters of around 4,500 persons each. This translates, approximately, to an area of 5 square kilometers based on the distance that can be covered by walking an hour.

### Chitral child survival program (CCSP) One step ahead

The CCSP trained 28 CMWs in partnership with the provincial and district MNCH program. These CMWs successfully cleared the exams and received degrees from Pakistan Nursing Council. While deploying these 28 CMWs, the national MNCH program guidelines were followed. For the poor and economically vulnerable families of Chitral, having access to CMWs at the community level has the potential to reduce out-of-pocket expenditures for travel and the opportunity costs associated with time spent away from work or home [7,8].

CCSP then built upon the national MNCH program by implementing an additional set of targeted activities that aim to embed CMWs into their communities by increasing the uptake of their services on a financially sustainable basis. Despite efforts to build up the cadre of CMWs, the GoP has not yet designed or considered a financial mechanism to sustain CMW services over time or to enable communities to pay for services. One of the primary objectives of CCSP is to decrease financial barriers to MNCH services, in particular those delivered by the CMW.

### Community-based savings groups (CBSGs)

To achieve financial sustainability for CMWs continued presence and accessibility to the community, CCSP established Community-Based Savings Groups (CBSGs). These CBSGs are composed of approximately 10 to 30 women who pool their savings and then lend out internally through group consensus at a pre-defined and mutually agreed upon interest rate. CBSGs are a simple, transparent, cost-effective and sustainable means of providing entry level financial services to the very poor and have been identified as a viable mechanism for poor rural populations that are most often neglected in formal microfinance systems that find it too risky and costly to provide credits to the poor living in remote villages [9]. CBSGs enable the poorest, most marginalized to participate in the community’s socio-economic development and also allow a platform for community interaction. CBSGs are self-elected and encourage participation of each household. All members of CBSGs are eligible to avail loans in emergencies [10]. CBSGs address traditional constraints to utilizing MNCH care through empowering them through economic means, as well as offering a platform for group solidarity and creating awareness on health seeking behaviors.

### Rationale and Aim of study

CBSGs are an innovation in Pakistan, and their use to reduce financial barriers specifically for MNCH services has not been tested globally [11]. The link between CBSGs and utilization of MNCH services is currently unknown. This study will attempt to answer whether membership in CBSGs contributes to increased awareness of service availability, understanding of MNCH issues, in addition to greater utilization of MNCH services in the community, specifically those offered by CMWs.

Initial evidence suggests that membership in CBSGs has improved the financial literacy, management capacity and economic power of women, which in turn has led to greater respect in the community, increased decision-making power, and the confidence to interact with influential figures, and participate in village assemblies/community institutions [12]. Through the qualitative methods, the associated pathways of financial autonomy, decision-making authority, level of empowerment, will be analyzed to further explain the financial and social effects of CBSGs and CMW service utilization.

This project holds promise to produce evidence supporting a model that will directly address the major obstacles to accessing skilled birth attendance i.e., distance, inability to pay, perception of risk for an obstetric emergency, traditional preference for home delivery and cultural barriers to women’s mobility outside the home. Given the results of these findings, CBSGs may be replicated in other comparable areas in Pakistan as a viable mechanism to increase MNCH service utilization amongst rural, poor, and marginalized communities.

The project is in line with the GoP’s Community Midwifery Initiative, National Health Policy 2010, the Benazir Income Support program (BISP) and other community based health financing and social protection schemes.

### Research hypothesis

The hypothesis of the study is ‘whether membership in CBSGs of women or of their family members is associated with greater utilization of MNCH services.’

### Primary objective

The primary objective of this study is to assess the relationship between the membership of a CBSG and the utilization of CMW MNCH services.

### Secondary objective

The secondary objectives of the study will address the following questions:

1. To what extent are financial services of CBSGs associated with utilization of CMW MNCH services?

2. To what extent are social benefits of CBSGs associated with utilization of CMW MNCH services?

This research question and sub-questions will determine whether there is an association between membership within a CBSG and the use of CMW MNCH services. These include: antenatal care, referrals to facilities, skilled delivery, and postnatal care. This question will be answered through a mixed methods study.

## Methods

### Study design and duration

The cross sectional study’s target population is women of reproductive age group (WRA) who delivered within the last month. The total duration of the study will be 16 months, with 9 months allocated for data collection.

### Study population

The study will involve interviews with all women who have delivered within the last month in the eligible clusters and who give consent to be interviewed.

### Eligibility criteria

All women in reproductive ages who reside in the catchment area of the CMWs during the study periods, have delivered within one month, and give consent are eligible to be part of the study.

### Outcome variable and covariates

The outcome variable will be utilization of CMW services disaggregated by 1+ ANC, 1+ PNC, skilled delivery, and utilization of the full continuum of care, defined as the combination of all three mentioned services.

The primary independent variable of interest will be participation in a CBSG. The confounding variables may include: literacy; age; household income; household occupation; average distance to CMW’s home; distance to both FLHCF and SLRF; support of the community; presence of other service providers of perceived comparable quality and/or cost, etc.

Other possible factors affecting outcome could be: i) poor and extremely poor families may not be able to participate and contribute to the CBSGs; ii) uneducated mothers and families may not perceive messages clearly; iii) families with strong cultural restrictions on female mobility may not utilize CMW services and follow her instructions for timely care FLHCF and SLRF; iv) natural hazards like floods, land sliding and snow avalanches that may block roads and affect referrals and v) intervention villages at greater distances and travel time may aggravate high risk pregnancies before reaching the SLRF.

### Sampling strategy

**Figure 1:**
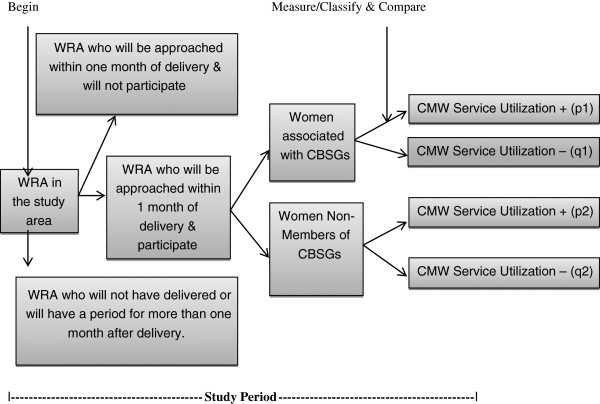
Flow chart of study.

The study will adopt purposive sampling in which all eligible women from the CMW’s surveillance area will be approached and interviewed after taking their consent during the study period. Out of 28 CMWS, we included 24 who were deployed in the community. 4 CMWs were excluded as they were not deployed. Refer to Figure 1 for the study participants and their distribution into groups.

### Sample size assumptions

According to the recent survey conducted by Benazir Income Support Program (BISP), the target intervention population of Chitral is 69841 [13]. In the 24 community clusters where CMWs had been deployed, 27% of the population consists of women in reproductive age. With the population growth rate of 3.3, it is expected that at least 2095 deliveries will be conducted in this population. Refer Table 1. We assumed that there will be at least 10 percent increase in the deliveries conducted by a SBA with the introduction of the CMWs in the communities (P1-P2 >10%). Refer Figure 1. The baseline survey indicates that 33% of all deliveries were conducted by SBA. We assume that skilled deliveries will increase from 33% to 40% in the absence of a CMW and further up to 50% with the introduction of CMWs. To measure this difference of at least 10%, with the average ratio of women participating in the CBSG in the population as 1:5, at 5% level of significance with power of 80% the sample size was calculated as 1160, which was further increased to 1218 women to account for non-response rate and refusals.

**Table 1:** Data used for sampling purpose

**Variable**	**Total**
CMWs deployed areas	24
Number of villages	219
Number of TBAs	71
Number of CHWs	32
Number of LHWs	89
Number of households (HH)	9042
Study population	69,841
Estimated number of women in reproductive ages	18,648
Estimated number of women who deliver per year	2,095

### Qualitative study

Focus Group Discussions (FGDs) will be conducted in 4 locations to capture diversity in geography, ethnicity and socio economic status within the program area. Further at each location, FGDs to be conducted with 4 groups comprising of the following categories:

1. Women who delivered within the last quarter and are member of CBSG

2. Men of the household where a women has delivered within the last quarter and is a CBSG member

3. Women delivered within the last quarter and are not a member of CBSG

4. Men of the household where a women has delivered within the last quarter and is not a CBSG member.

Individual cases will be randomly selected from the target population and followed throughout the duration of the study to capture changes in social and financial factors that shape health behavior and decision making over time in the form of case studies.

### Data collection

A data collector will be hired in each community cluster that will be responsible to identify, in collaboration with CMWs, TBAs and LHWs, all the women who have recently delivered in the area. After taking an informed consent, data will be collected from the eligible women through a structured closed-ended questionnaire.

The questionnaire will have five sections: Socio demographic and economic characteristics, knowledge about CMWs and their utilization, information about previous pregnancy, information about previous delivery and post natal care, and participation and utilization of CBSG. Considering that the participants are women who recently delivered, the questionnaire is short, concise and with relevant questions only.

For the qualitative component, women and men (according to the criteria mentioned in the sampling strategy) in the selected community clusters will be invited to participate for a FGD. Each group will comprise of 6–10 eligible men or women. The FGD will be following the grounded theory with constant comparison technique and will be according to the FGD tool developed for each of the particular group. The FGD will be terminated at the point of saturation.

To maintain confidentiality in responses, each interviewee will be assigned a unique ID number and will be interviewed on an individual basis without other members of the household present.

### Data management

Data collected by the data collector in the community will be assessed for completion and errors at the field level by the collectors. Then the questionnaire will be submitted to the field supervisor for logical error checking and completeness. In addition to this 5% of the questionnaire will be re-filled by the supervisor to ensure correctness and validity of the responses.

Questionnaire will then be provided to data management officer by field supervisors for double data entry into the database. 10% of the entered data from each batch will be checked for data entry errors by data management officer. The particular batch of questionnaire will be reentered if the accuracy in data entry in any batch is found to be less than 99%. Furthermore, the technical team will check 10% of the data to ensure proper quality and validity.

### Data analysis

This study aims to analyze the association of CMW services amongst women who are associated with a CBSG and those who are not. Descriptive statistics will be computed: proportions for categorical variables, mean and standard deviation for continuous variables having normal distribution, median and inter quartile range for continuous variables having skewed distribution.

Chi-squared test will be used to determine the difference in proportion and the relationship between CMW utilization and CBSG membership .The quantitative data will be analyzed through logistic regression model with outcome variable as utilization of CMW services as dichotomous, and the primary independent variable as membership of CBSG also as dichotomous. All confounders and effect modifiers will be adjusted for in the regression models.

The data from the FGDs will be translated to English and Khowar - a local language of Chitral district. The qualitative data will be analyzed manually by identifying nodes and sub-nodes to generate themes. Furthermore, the extent to which financial or social empowerment through a CBSG leads to greater utilization of MNCH CMW services will be analyzed in this study. Pathways that may lead to utilization of CMW services include: greater financial access, increased knowledge and acceptance of CMW services, enhanced confidence, greater decision-making power within the household, etc. Additionally, given that women may be associated with a CBSG through another member of the household, as opposed to being members themselves, analysis will be conducted to determine whether there are, within these two categories, different levels of perceived status, decision-making in the household, or sense of empowerment by women, and how these factors may have shaped their decisions during pregnancy.

The FGD of male members of the household of all women who have delivered with one month will be aimed at assessing the socio-cultural issues associated with decision-making patterns for MNCH services and choice of provider, perceived level of empowerment of marginalized women in the community, impact of the CBSG in financial access, awareness and knowledge of available MNCH services, perception of CMW and awareness of high risk pregnancies. The FGD of women who are members of a CBSG and who belong to a household of an interviewed woman who has delivered within one month will generate information about factors that have influenced the reasons for joining the saving group, the purpose of availing loans, and the relative decision-making power she holds within the household.

Data collected from CBSG managers will provide information regarding purpose and utilization of CBSG funds, priorities of CBSG members that require them to access loans. Qualitative analysis of the interviews, FGDs, and case studies will be conducted with the software ATLAS TI.

### Monitoring and evaluation

Quality assurance will be an integral part and key focus throughout the project. Routine data quality assessments mechanisms to ensure data quality include sample accuracy checks in data collection and data entry. In addition, interim reviews will also be conducted to ensure the effectiveness of overall data quality assessment process and data entry.

Capacity building on monitoring and evaluation tools, processes and procedures will be done at the initial stage of the project. This will ensure that the project team members at all levels gain access to M&E information and receive feedback for effective decision making throughout the project. Further, these tools, process and procedures will generate consolidated information for project stakeholders and other interested groups. Tracking lessons and best practices will also be incorporated into the system so that any interest groups can access to these information and utilize them to design similar research studies in the future.

### Dissemination

This research can help identify whether CBSGs have the capacity to reduce financial barriers to CMW services – a key issue which is not adequately addressed in the national MNCH program. CBSGs, if proven successful, have the potential to reduce 2 of the 3 delays of maternal deaths. In order to ensure that the findings from this research are disseminated in an effective manner to all the important audiences and stakeholders, multi-level dissemination strategy will be used. Seminars will be conducted at national, district and community level with relevant stake holders. Additionally, communities will also be made aware of the research and its findings through local newspaper and radio stations. National newspapers will also be used to reinforce the concept and also to reach to the larger audiences e.g. influencers, pressure groups, social development NGOs and policy makers. Research papers will be presented at relevant international and national conferences, and a manuscript will be submitted for publication in international and national journals.

## Discussion

### Limitations of the study

1. Contamination due to shared roles of CMWs and TBAs: CMWs are young women who have been trained recently and were introduced into the community as a new cadre of community health care provider. They are competing with traditional birth attendants into the community. Although a BCC campaign has been initiated to introduce the CMWs, clarify their roles, and link them with TBAs for certain activities, it is, nevertheless, presumed that communities may have different responses to CMWs and this will have an impact on utilization of their services.

2. Temporal trend: CMWs have just been deployed into their respective communities with few months of interaction considering the dynamics of the village communities and the cultural barriers that exist therein, it is assumed that CMWs will take time setting in. Utilization of CMW services is thus may not be consistent during the entire length of the study period with utilization in the initial period far less than later phases.

3. Women’s priorities to use CBSG fund: It is possible that women in Chitral may have priorities other than health and need to utilize CBSG funds in other areas as compared to MNCH services. This will lead to competing interests from multiple exposures, which will later act as a confounding factor for the outcome.

4. Enrolment of pregnant women: There is a possibility that the deliveries may be missed during the data collection process. This can lead to volunteer bias; those who enrolled for the project may be having different baseline characteristics from those who did not enroll in the project.

5. Cultural barriers: In some communities, cultural barriers may affect mobility in some of the villages. Enrolment of poor and ultra-poor households as member of the CBSGs may be limited due to their inability to pay the monthly contribution amount and to purchase shares.

### Ethical considerations

All interviews will be conducted only after receiving informed consent. Field-based data collectors/enumerators will seek consent from each participant before conducting interviews and FGDs. Additional consent will be required for those included in case studies in this research. Informed consent will describe the risks and benefits involved in the study and will explain what is required of each subject. The study has been approved by the National Bioethics Committee (NBC) of Pakistan.

There are no major ethical considerations. However, if a data collector finds out that a woman being interviewed is suffering from some complication as a result of the last pregnancy, she will refer this patient to the CMW in the area. This will not have any impact on the study results as this referral will be done after the data collection process.

### Gender, poverty, equity, and social inclusion

This study seeks to address the relative impact of CBSGs on utilization of CMW MNH services in the most remote and marginalized populations of Chitral. Furthermore, the design of this study enables comprehensive representation of the target population – women of reproductive age who have delivered within one month and who may or may not have accessed CMW services throughout their pregnancy, or may or may not be a member of a CBSG. Because women require any amount of cash to join a CBSG, it is acknowledged that CBSGs may exclude the poorest individuals in the community. Furthermore, given the social cultural norms in Pakistan, any form of gathering is almost always segregated between men and women. Though CBSGs only currently consist of women in CCSP CMW areas, men may also choose to form their own CBSGs in future. The study design allows capturing all eligible women in the study population, the associated male members in the women’s households, and the managers associated with the CBSGs.

### Environmental considerations

Given the harsh weather conditions, particularly during the winter months, weather may prove to be a limiting factor for adhering to the data collection schedule. Because data collectors/enumerators are based in the field, it should help mitigate this challenge. During winter, heavy snow fall and avalanches may affect women access to MNCH services with CMWs and at FLHC and SLR facilities in case of an emergency.

## Competing interests

The authors declare that they have no competing interest (financial or otherwise) in this publication.

## Authors’ contributions

QN will be responsible in providing overall guidance on the research design, implementation, and analysis. He will ensure that the research meets its objectives, routinely analyze study findings, identify issues and develop mitigation measures. IA will perform the sample size estimation, data management and statistical analysis. BTS to review the qualitative component of the research design, develop detail implementation plan, provide training of supervisors in the field. TR will facilitate the data management and quality assurance during the study period. SA will coordinate the proposal development, implementation and maintaining compliance with donor requirement. SW will coordinate the study implementation and dissemination with local stakeholders. PR will work alongside CCSP to develop design and concept of CBSGs within the program area. He will also provide technical guidance on research design, implementation, and analysis. WJ will be involved in reviewing the manuscript. All the authors will approve the final manuscript.

## Pre-publication history

The pre-publication history for this paper can be accessed here:

http://www.biomedcentral.com/1471-2393/13/185/prepub

## Implementing partners names

AKFP will be the lead executing agency for this research and will maintain close coordination with AKHSP and AKRSP throughout the study duration. As part of the CCSP, AKHSP is primarily responsible for training, deployment, and supervision of CMWs in Chitral, while AKRSP is responsible for formation and facilitation of CBSGs through social mobilization. All costs associated with the CCSP project/intervention implementation and management will continue to be borne by the USAID grant. The only costs associated with the RAF proposal are those directly related to this research study.
